# First Report on Antifungal Activity of *Metschnikowia pulcherrima* Against *Ascosphaera apis,* the Causative Agent of Chalkbrood Disease in Honeybee (*Apis mellifera* L.) Colonies

**DOI:** 10.3390/jof11050336

**Published:** 2025-04-25

**Authors:** Massimo Iorizzo, Francesca Coppola, Gianfranco Pannella, Sonia Ganassi, Cristina Matarazzo, Gianluca Albanese, Cosimo Tedino, Licia Maria Di Donato, Vincenzo Pio Iacovino, Rosaria Cozzolino, Antonio De Cristofaro

**Affiliations:** 1 Department of Agriculture, Environmental and Food Sciences, University of Molise, 86100 Campobasso, Italy; iorizzo@unimol.it (M.I.); sonia.ganassi@unimol.it (S.G.); c.matarazzo3@studenti.unimol.it (C.M.); cosimoted_91@libero.it (C.T.); l.didonato@studenti.unimol.it (L.M.D.D.); v.iacovino1@studenti.unimol.it (V.P.I.); decrist@unimol.it (A.D.C.); 2 Department of Agricultural Sciences, University of Naples “Federico II”, 80055 Portici, Italy; francesca.coppola2@unina.it; 3 Department of Science and Technology for Sustainable Development and One Health, University Campus Bio-Medico of Rome, 00128 Rome, Italy; g.pannella@unicampus.it; 4 Institute of Food Science, National Council of Research (ISA-CNR), 83100 Avellino, Italy

**Keywords:** chalkbrood, *Apis mellifera*, *Ascosphaera apis*, Metschnikowia pulcherrima, volatile organic compounds (VOCs), biocontrol

## Abstract

Chalkbrood is the manifestation of the fungal disease caused by *Ascosphaera apis*, which affects broods of developing honeybees, particularly in *Apis mellifera* colonies. Recently, *Metschnikowia pulcherrima* has been proposed as a biocontrol agent in winemaking and for the management of major postharvest and soil-borne plant pathogenic fungi. In this study, the antagonistic activity of three *M. pulcherrima* strains against fifteen *A. apis* strains, isolated from contaminated hives of *A. mellifera*, was evaluated, with a specific focus on the potential antifungal activity of volatile organic compounds (VOCs). The study revealed that *M. pulcherrima* was effective against *A. apis* and that the antifungal activity was related to various mechanisms including competition for nutrients, secretion of pulcherriminic acid, and biosynthesis of specific antifungal VOCs. We also found that each *M. pulcherrima* strain produced a unique combination of VOCs, and the antifungal activity was strain-dependent and varied depending on the specific yeast-mold combination. In addition, preliminary analyses showed that a temperature of 30 °C and a higher amount of glucose (40 g/L) in the growing medium promote the growth of *A. apis*. These results could be useful for designing new strategies for the biocontrol of chalkbrood disease in honeybee colonies.

## 1. Introduction

Pollination is an ecosystem function considered fundamental to plant reproduction, agricultural production, and the maintenance of terrestrial biodiversity [1,2].

A loss of pollinators may have negative impacts on the reproduction of wild plants, as more than 90% of tropical flowering plant species and about 78% of temperate-zone species rely, at least in part, on animal pollination [3]. The potential risks associated with pollinators’ decline have led to a surge in research aimed at understanding the factors and interactions that are crucial to developing targeted responses to this pressing issue [4,5]. There are over 20,000 species of bees worldwide, which represent the dominant pollinators in most ecosystems. Specifically, the western honeybee, *Apis mellifera*, is the most ubiquitous managed crop pollinator worldwide [6].

In the past five decades, there has been a documented decrease in the biodiversity of wild bees and other pollinators [7]. The factors deemed responsible for this decline include land-use change and intensity, climate change, pesticides, and the transfer of pathogens and diseases [8]. Recent studies have demonstrated that stress factors do not act in isolation but rather synergistically and that their interactions can be difficult to predict [9]. While pathogens and diseases primarily threaten managed European honeybees [10], the decline in wild bees seems to be particularly related to land use change and agricultural intensification [11,12,13,14].

A honeybee colony can host a diverse range of pathogens, including bacteria, fungi, viruses, parasitic mites, and even insects that aim to exploit the abundant resources within the hives, which contributes to colony decline [1,15,16]. Chalkbrood, caused by the fungus *Ascosphaera apis*, is the common name for a fungal disease that weakens developing broods and impacts various bee taxa, particularly *A. mellifera* colonies [17,18,19,20]. Recent evidence suggests that the incidence of this disease is globally on the rise [21,22,23,24], probably attributable to the wide distribution of *A. apis* and the prolonged viability of its spores [25]. Recent research shows that chalkbrood makes honeybees more vulnerable to other pests and diseases, such as viruses that might infect and replicate within *A. apis* [26,27,28]. Clinical outbreaks of chalkbrood are primarily caused by environmental factors, the colony’s health status, genetic predispositions, and the stress factors affecting the brood at the time of infection [29,30]. The initial phase of infection can be facilitated by nutritional or environmental stressors that disrupt the composition of the gut microbiota [31]. Khan et al. [32] suggested that a decline in culturable aerobic gut bacteria in nurse bees serves as a prognostic marker for chalkbrood outbreaks. In this context, bacteria from the bee gut that inhibit chalkbrood have been proposed as potential probiotics for controlling chalkbrood and other fungal diseases in bees [32,33,34]. Honeybee larvae aged 1 to 4 days are particularly susceptible to fungal infection by *A. apis*, which is primarily transmitted through contaminated food provided by nurse bees. Infection occurs when larvae ingest ascospores orally, leading to spore germination in the posterior midgut. The fungal hyphae invade the epithelial cells and basal membrane of the midgut, with the mycelium then progressing into the hemocoel and eventually penetrating all organs, causing larval death by day 3 [35]. Over the past years, a wide range of chemotherapeutic compounds have been tested against *A. apis* [29,33], but none have proven effective in preventing the disease. The human health risks associated with pesticide and antifungal residues in honey have driven growing interest in natural compounds as alternative control strategies [36,37,38,39,40,41].

The use of microbial resources for the prevention and biocontrol of honeybee pathogens, including chalkbrood disease, presents promising possibilities [42,43,44]. Iorizzo et al. [33] demonstrated that *Apilactobacillus kunkeei* (formerly *Lactobacillus kunkeei*) and *Lactiplantibacillus plantarum* [34] inhibit the mycelial development of *A. apis* in vitro. These bacteria may help restore gut symbiosis and prevent *A. apis* infection. Additionally, studies indicate that beneficial bacteria isolated from the bee gut and hive products show potential as probiotics for controlling chalkbrood disease [21,43,45]. Over the past decade, researchers have increasingly focused on the role of the honeybee gut microbiome, investigating its specific microbial components [46,47,48,49]. While most studies on insect-associated microbiota have concentrated on bacteria, other microbial partners, such as fungi, have been largely overlooked. Insects frequently host microbial symbionts, with insect-fungal associations playing key roles in nutrition and defense [50]. Within the gastrointestinal tract of the insects, including honeybees, there is also an eukaryotic portion represented by yeast species [51,52,53]. Yeasts have been identified in at least 143 insect species across various orders, indicating their widespread and diverse associations with insects [52,54]. Although symbiotic relationships between hymenopterans and fungi have been documented, and yeasts are known to be abundant in bee bread and nectar, the honeybee mycobiome and its impact on host fitness remain largely unexplored. Recently, probiotics—particularly lactic acid bacteria (LAB) and select yeasts—have gained attention as promising alternatives to antibiotics for managing bee diseases [43,44,55,56]. The most commonly found yeast genera in the honeybee gastrointestinal tract and its agro-ecosystem (including bee bread, propolis, and pollen) include *Candida, Debaryomyces, Metschnikowia, Meyerozyma, Pichia, Starmerella*, and *Zygosaccharomyces* [55,56,57]. Most *Metschnikowia* species (and anamorphs) are terrestrial and form diverse mutualistic symbioses that include a multitude of associations, especially with angiosperms and their associated insects [58]. The strong antagonistic properties of *M. pulcherrima* have sparked research into its ecological roles, yet a comprehensive understanding of its interactions and their modulation by environmental factors remains limited [59]. Recently, *M. pulcherrima* has gained interest as a potential probiotic yeast for animal and human feed [55,60,61,62,63,64,65]. Although the nature of the association between *Metschnikowia* species and insects is not fully understood, evidence suggests that these relationships can be highly specific [66]. Due to its strong antimicrobial activity and the absence of toxic byproducts, *M. pulcherrima* has been the focus of several technological innovations. Over the past decade, multiple patents have been filed concerning its applications [67,68], and numerous *M. pulcherrima* strains have been proposed as biocontrol agents in winemaking, cheese production, and the management of major postharvest and soil-borne plant pathogenic fungi [69,70,71,72,73,74,75,76,77,78,79,80,81,82].

The antimicrobial activity of *M. pulcherrima* is mediated by multiple mechanisms, including iron depletion through pulcherrimin production, competition for nutrients, secretion of extracellular lytic enzymes (e.g., chitinase and glucanase), biofilm formation, and the release of volatile organic compounds (VOCs) [67,77]. In particular, previous studies have shown that among the main VOCs released by *M. pulcherrima*, ethyl acetate and 2-phenylethanol are effective in postharvest preservation of strawberries and blueberries [71,83].

To date, no scientific studies have explored the use of *M. pulcherrima* against pathogenic honeybee fungi, particularly *A. apis*. Therefore, this study aimed to assess the antagonistic activity of three previously isolated and characterized *M. pulcherrima* strains [84,85,86] against fifteen *A. apis* strains obtained from contaminated hives. Antimicrobial tests were conducted using different cultural matrices of the three mentioned yeasts, with a specific focus on evaluating the antifungal efficacy of VOCs produced by them.

## 2. Materials and Methods

### 2.1. Yeast Cultures

*M. pulcherrima* 62, *M. pulcherrima* 86, and *M. pulcherrima* AS3C1 (GenBank accession numbers: PP922572.1, PP922571.1, OM038321) were obtained from the culture collection of the Di.A.A.A. (Department of Agricultural, Environmental, and Food Sciences, University of Molise, Campobasso, Italy).

### 2.2. Isolation of Ascosphaera apis

In *A. mellifera* colonies, the presence of mummified larvae in the brood comb, lower hive table, and hive entrance has been considered a common sign of chalkbrood disease. The fungal strains used in this study were isolated from fresh white mummies collected during the springs of 2022 and 2023 from brood frames, bottom boards, or hive entrances in the Campania and Molise regions of Southern Italy.

**Figure 1:**
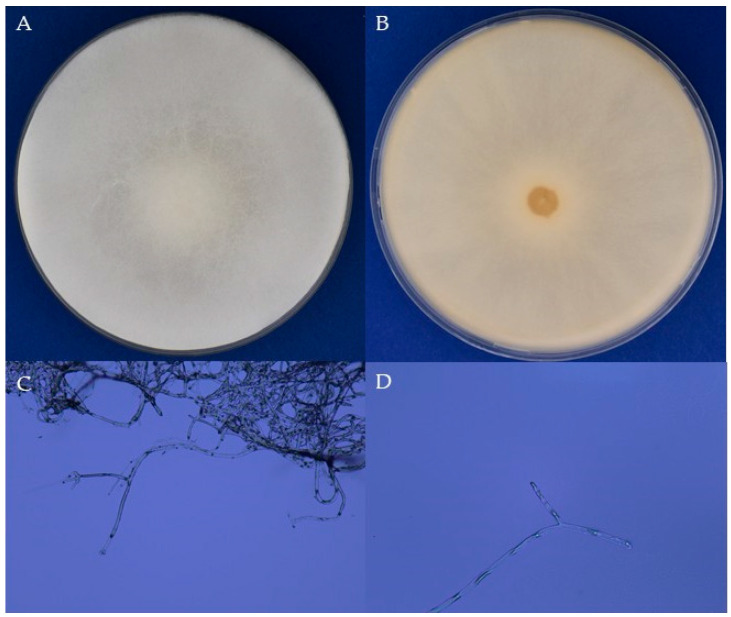
Morphological characters of the colony ((**A**) front; (**B**) back) and mycelial hyphae ((**C**) 100×, (**D**) 200×) of *A. apis* CB3 on SDA.

Following the method described by Jensen et al. [87], the collected mummies were surface-sterilized in 10% sodium hypochlorite for 10 min and then rinsed with sterile distilled water for 2 min under aseptic conditions. The mummies were subsequently dissected into smaller fragments and placed on Sabouraud Dextrose Agar (SDA) plates [87]. Once mycelial growth became visible around the inoculated samples, incubated at 30 °C for 3–4 days, actively growing fungal tips were transferred onto fresh SDA plates. The purification of *A. apis* was achieved through three rounds of subculturing of hyphal tips on SDA according to the methods described by Jensen et al. [87]. All strains have the same morphological characteristics. By way of representation, Figure 1 shows some morphological details of the colony and mycelial hyphae of *A. apis* CB3 on SDA.

### 2.3. Genotypic Identification

Molecular identification of the fungal cultures was conducted through sequencing and analysis of the internal transcribed spacer (ITS) region of nuclear rDNA. Genomic DNA was extracted from the mold strains using Fungi/Yeast Genomic DNA Isolation Kits (Norgen Biotek, Thorold, ON, Canada), following the manufacturer’s instructions.

The full ITS region was amplified using the universal primers ITS1 (5′-TCC GTA GGT GAA CCT GCG G-3′) and ITS4 (5′-TCC TCC GCT TAT TGA TAT GC-3′) [88]. The polymerase chain reaction (PCR) mixture consisted of 10 μL of 2× PCR master mix (Norgen Biotek), 2 μL of each primer (2.5 μM), 4 μL of Milli-Q water, and 2 μL of template DNA. For the negative control, Milli-Q water was used as a substitute for the DNA template. PCR reactions were conducted using a Mastercycler Nexus PCR thermal cycler (Eppendorf, Hamburg, Germany). The amplification process followed a thermal cycling program consisting of an initial denaturation at 95 °C for 10 min, followed by 35 cycles of denaturation at 95 °C for 30 s, annealing for 1 min, and extension at 72 °C for 1 min. A final extension step was performed at 72 °C for 5 min. The PCR products were analyzed using electrophoresis on a 1.0% (*w/v*) agarose gel in a 1× Tris-acetate–EDTA (TAE) buffer. The amplified DNA was visualized under a UV transilluminator (Bio-Rad, Hercules, CA, USA), and fragment sizes were estimated by comparison with a 1 kb DNA ladder (Norgen Biotek). The PCR products were purified using a QIAquick PCR purification kit (QIAGEN GmbH, Hilden, Germany) and subsequently sent to an external sequencing service (Eurofins MWG Biotech Company, Ebersberg, Germany). The resulting sequences were analyzed using the Basic Local Alignment Search Tool (BLASTN) program [89] and compared with reference data available in the National Center for Biotechnology Information (NCBI) database [90]. A sequence alignment of 99–100% similarity was considered the criterion for taxonomic identification at the species level [91].

A phylogenetic tree was inferred using the ITS sequences of the fifteen *A. apis* strains with the maximum likelihood method. Genetic distances were calculated based on the Kimura 2 parameter model [92] by applying 1000 replicates. Evolutionary rate differences among sites were modeled using a discrete gamma distribution (5 categories, +G, parameter = 0.2275). Evolutionary analyses were performed in MEGA11 [93].

### 2.4. Growth Parameters of A. apis

Three culture media, potato dextrose agar (PDA), Sabouraud dextrose agar (SDA), and malt extract agar (MEA), were used for fungal growth experiments (Thermo Fisher Scientific, Waltham, MA, USA).

Three-day-old cultures of each *A. apis* strain were used for the tests. A single disk, excised from the edge of actively growing mycelium, was placed at the center of each Petri dish (90 mm) containing the respective medium. All plates were incubated at three different temperatures (25.0 ± 1.0 °C, 30.0 ± 1.0 °C, and 35.0 ± 1.0 °C), with relative humidity (RH) maintained at approximately 60%. After six days of incubation, the mycelial growth was assessed by measuring the colony diameter (mm) using a caliper.

### 2.5. Antifungal Activity

Different methods were used to investigate the antifungal activity of *M. pulcherrima* against *A. apis.*

#### 2.5.1. Preliminary Antifungal Screening

The antifungal activity of *M. pulcherrima* against *A. apis* was assessed following the protocol described by Iorizzo et al. [33] utilizing three distinct matrices: broth culture (BC), cell-free supernatants (CFS), and cell pellet (CP). To obtain these fractions, *M. pulcherrima* was cultured in yeast peptone dextrose (YPD) broth (Thermo Fisher Scientific) and incubated at 30 °C for 48 h, achieving a final cell concentration of 10⁸ CFU/mL. The untreated yeast culture served as the BC matrix. To prepare the CFS fraction, 5 mL of the yeast culture were centrifuged at 8000 rpm for 15 min at 4 °C, and the resulting supernatant was sterilized via filtration using a 0.22 μm pore-size cellulose acetate filter. The CP fraction was obtained by washing the residual cell pellet and resuspending it in 5 mL of sterile distilled water. Antifungal assays were performed by inoculating single mycelial discs (6 mm in diameter) of *A. apis*, pre-cultured on SDA for three days at 30 °C, into the center of 90 mm Petri dishes containing the SDA medium. Each plate was supplemented with 5 mL of one of the *M. pulcherrima* matrices (BC, CFS, or CP). A control test was also included, consisting of an *A. apis* mycelial disc on SDA without yeast-derived matrices. All plates were incubated at 30 °C under aerobic conditions, with three biological replicates per yeast-fungus combination. After six days of incubation, the radial growth of *A. apis* mycelium was measured using a digital caliper and compared against the control test. The inhibition of radial growth (% I) was determined using the equation *%* I = (C − T/C) × 100 [94], where % I represents the percent inhibition of radial mycelium growth, C is the radial growth measurement in the control test, and T corresponds to the radial growth of *A. apis* in the presence of *M. pulcherrima* matrices (BC, CFS, and CP).

#### 2.5.2. Evaluation of the Antifungal Activity of *M. pulcherrima* VOCs

**Figure 2:**
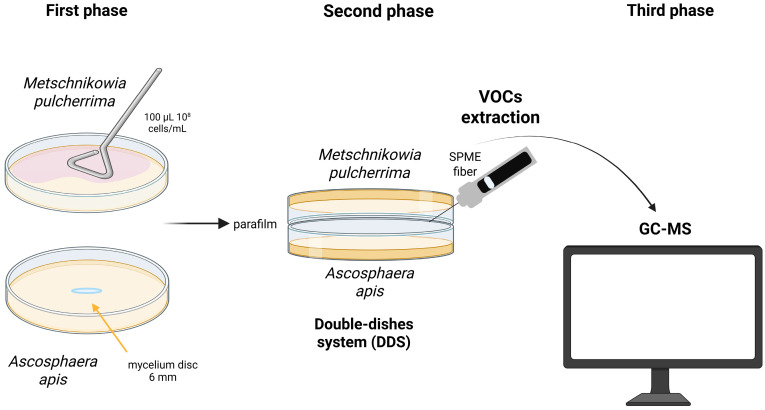
Experimental workflow for the double-dishes system (DDS) used to evaluate the antifungal activity of *M. pulcherrima* volatile organic compounds (VOCs). Phase 1: *M. pulcherrima* was cultured on YPD agar medium, while *A. apis* was grown on SDA medium under standard incubation conditions. Phase 2: The two Petri dishes were sealed with Parafilm, positioned face-to-face to establish the DDS, and incubated for 4 days to allow VOC emission; VOCs were subsequently captured using a solid-phase microextraction (SPME) fiber. Phase 3: VOCs were analyzed using gas chromatography coupled with mass spectrometry (GC-MS) for qualitative and quantitative profiling (created with BioRender.com, https://app.biorender.com/illustrations/67c1d80f4c97b8e297f2f262, accessed on 10 March 2025).

The inhibitory effect of volatile organic compounds (VOCs) produced by *M. pulcherrima* against *A. apis* was assessed using a double-dish system (DDS), following the methodology described by Ruiz-Moyano et al. [95] with modifications (Figure 2). A 100 μL aliquot of a 48 h *M. pulcherrima* culture, standardized to 1 × 10⁸ cells/mL in YPD broth, was evenly spread onto YPD agar plates (90 mm). Concurrently, a single *A. apis* mycelial disc (6 mm in diameter), pre-cultured on SDA for three days at 30 °C, was placed at the center of an SDA plate. The lids of both plates were removed, and the two dishes were sealed together using Parafilm (Pechiney Plastic Packaging Company Milwaukee, WI, USA) to create a closed DDS chamber. The system was incubated at 30 °C, with the *A. apis*-inoculated plate positioned at the bottom. Fungal radial growth was measured daily using a digital caliper. The control consisted of a DDS setup containing only *A. apis*. After six days of incubation, radial growth inhibition was calculated using the previously described formula. Each yeast-fungus combination was tested in triplicate.

### 2.6. Identification of Volatile Organic Compounds (VOCs)

Antifungal VOCs were identified using *M. pulcherrima* strains AS3C1, 86, and 62 as producer strains, with *A. apis* CB3 serving as the target fungal strain. All tests were conducted in triplicate.

#### 2.6.1. Volatile Compounds Extraction

VOCs were extracted by headspace solid-phase microextraction (HS-SPME). According to Ruiz-Moyano et al. [95], to sample the VOCs, the double-dish system (Figure 1) was previously incubated at 40 °C for 15 min in an oven. Successively, a DVB/CAR/PDMS fiber (50/30 mm, 2 cm) was introduced into the space of the double-dish system for 30 min at 40 °C and then inserted into the GC injector to allow the VOCs’ desorption at 240 °C for 10 min. Volatile compounds were determined by extraction and analyses of control DDS without yeast inoculation. Blank runs were also run to avoid possible volatile contamination during the analysis.

#### 2.6.2. Gas Chromatography/Mass Spectrometry (GC/MS) Analysis

The VOCs were analyzed by an Agilent 7890A gas chromatographer (7890A, Agilent Technologies, Santa Clara, CA, USA) hyphenated to a 5975A mass spectrometer (5975A, Agilent Technology, Santa Clara, CA, USA) and equipped with a capillary column HP-Innowax (30 m × 0.25 mm × 0.5 µm). Analyses were performed as described by Serradilla et al. [96]. In particular, the oven temperature was initially set at 40 °C for 3 min, followed by a temperature increase to 150 °C at a ramp rate of 4 °C/min holding for 1 min and a second increase to 150 °C at a ramp rate of 3 °C/min, holding the final temperature for 2 min. Helium, at a flow rate of 1 mL/min, was the carrier gas. The temperatures of the ion source and quadrupole were 230 °C and 150 °C, respectively. Mass spectra were carried out in electronic impact (EI) mode at 70 eV, using the splitless mode and in the range of m/z 30–300.

The mass spectra libraries Nist05 and Wiley07 and the linear retention indices (LRI) were used for VOCs identification. Moreover, the identity of certain compounds was confirmed by using commercial standards analyzed under the same conditions. Semi-quantitative data of each volatile metabolite (relative peak area, RPA%) were measured as a ratio for the peak area of 3-octanol used as an internal standard (IS). Areas of the identified VOCs were obtained from the total ion chromatogram (TIC).

### 2.7. Statistical Analysis

The preliminary antifungal and antifungal activity of *M. pulcherrima* VOCs was evaluated through statistical analysis conducted in RStudio (R version 4.3.0). Data from three independent experiments were expressed as mean ± standard deviation (SD) and analyzed using ANOVA with Tukey’s post hoc tests. Statistical significance was attributed to *p*-values < 0.05. Additionally, VOC data were analyzed via principal component analysis (PCA) using the FactoMineR and factoextra packages (version 4.2.2) for data processing and visualization.

## 3. Results and Discussions

### 3.1. Taxonomical Identification

**Figure 3:**
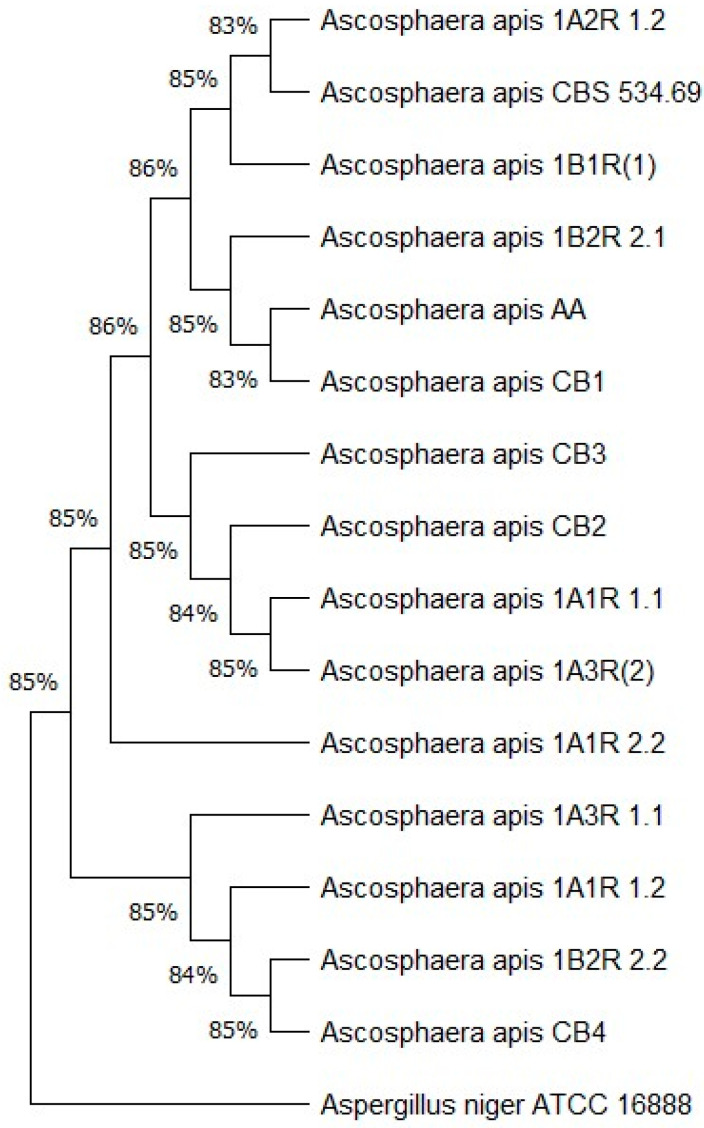
Phylogenetic tree based on the ITS sequences of the fifteen *A. apis* strains using the maximum likelihood method. The genetic distances were calculated based on the Kimura 2-parameter model by applying 1000 replicates.

The utilization of Sanger sequencing of the internal transcribed spacer (ITS) allowed for the identification of the 15 isolates as *A. apis*. The GenBank accession numbers for the sequences are provided in Table S1 (Supplementary Materials). The resulting phylogenetic tree (Figure 3) demonstrated that the isolates constituted a monophyletic group, suggesting the presence of a shared progenitor among the strains [97]. The percentages above the tree branches represent the site coverage. The presence of subclusters signifies the existence of genetic diversity, which can be attributed to either environmental adaptation, geographical origins, or host interaction. Some strains exhibited greater kinship, suggesting recent divergence, whereas others demonstrated a higher degree of genetic distance. *Aspergillus niger* was included as an outgroup alongside the *A. apis* type strain (CBS 534.69) to root the phylogenetic tree and emphasize the evolutionary divergence between *A. apis* and the fungal isolates.

### 3.2. Growth Parameters of the A. apis

**Figure 4:**
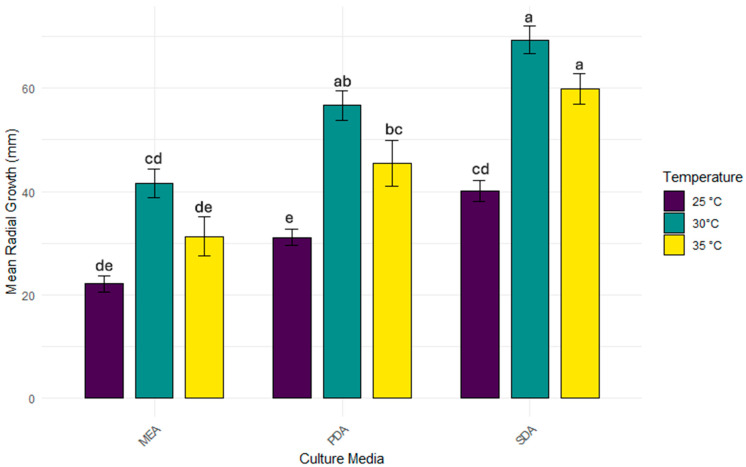
Mean radial growth (mm) after five days of the fifteen *A. apis* strains on different culture media (MEA, PDA, SDA) at 20 °C, 30 °C, and 35 °C. Error bars indicate the standard deviation (SD) of the mean. Lower case letters indicate statistical difference (*p* < 0.05).

Following taxonomic identification, a series of growth assays were performed under varying temperature conditions and across different agarized media. The data shown in Figure 4 reveal a clear trend in which temperature and culture media composition significantly influence fungal growth dynamics. Across all tested conditions, 30 °C appears to be the optimal temperature, supporting the highest radial expansion in all media. Growth at 20 °C and 37 °C is generally reduced. In the growth evaluation test on different culture media, *A. apis* strains exhibited more abundant radial mycelial growth on SDA, which contained a higher glucose concentration (40 g/L), compared to the other media used (Supplementary Materials Table S2), after five days. In Figure 3, it is possible to observe the radial mycelia growth of the fifteen *A. apis* strains as a function of temperature and culture medium used.

Our study confirms that media composition, particularly sugar content, critically influences the growth and development of *A. apis* [98,99]. Conversely, media high in fat or nitrogen content do not support the optimal proliferation of the pathogen [100]. The optimal growth temperature for the isolates was determined to be 30 °C, a finding that is consistently supported by the literature [98,100,101]. Honeybee colonies function as “superorganisms”, with individuals collaborating to ensure colony reproduction, particularly through social cooperation in thermal homeostasis, maintaining rapid and consistent brood development [102]. Thermal homeostasis is crucial for the colony, as honeybee larvae and pupae are highly stenothermic. Maintaining a brood nest temperature of 32–36 °C ensures rapid and consistent developmental rates [102]. *A. apis* is an opportunistic pathogen that infects and kills larvae only under predisposing conditions, with chalkbrood disease being more prevalent in damp weather and fluctuating temperatures [98].

In the context of colony dynamics, robust colonies capable of maintaining stable internal hive temperatures may better mitigate fungal proliferation. In contrast, weaker colonies struggling with thermoregulation will likely experience a more pronounced fungal spread. Flores et al. [103] demonstrated in a laboratory-scale study that maintaining a hive temperature of 35 °C correlates with a reduced infection rate. This may be explained by the fact that temperatures lower than the optimum can impair vital physiological processes, negatively impacting brood development and rendering the colony more susceptible to pathogen invasion [104,105].

### 3.3. Preliminary Antifungal Screening and Evaluation of the Antifungal Activity of M. pulcherrima VOCs

The preliminary evaluation of the antimicrobial activity of the three matrices (BC, CP, and CFS) obtained from the three yeasts against the 15 molds showed varying levels of intensity depending on the matrix and the yeast strain. The CP and CFS matrices caused 100% and 0% inhibition for all tested molds and yeasts.

**Figure 5:**
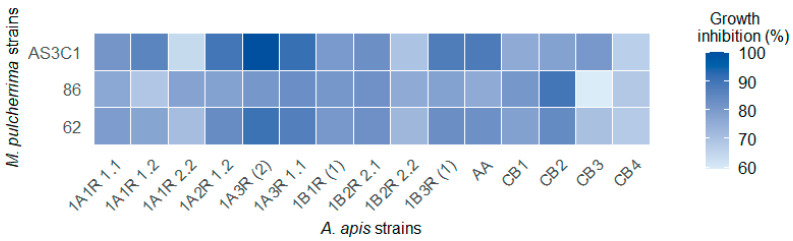
Heatmap showing the inhibition (%), after six days on SDA medium against fifteen *A. apis* strains caused by broth cultures (BC) of *M. pulcherrima* AS3C1, 86, 62.

**Figure 6:**
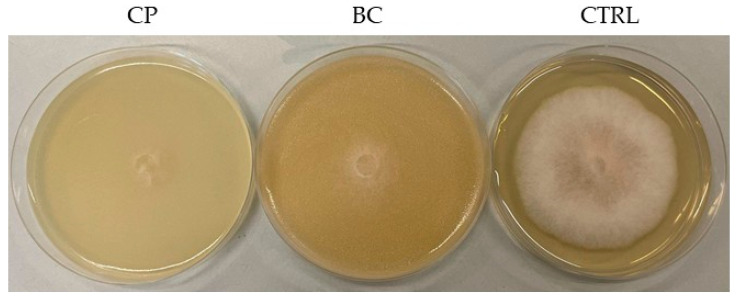
Inhibitory effect of CP and BC matrices of *M. pulcherrima* 86 on the mycelial growth of *A. apis* CB2 (CP: cell pellet, BC: broth culture, CTRL: control).

In the case of BC matrices, however, the percentage inhibition varied depending on the yeast-mold combination (Figure 5; Supplementary Table S3). On average, the BC with the highest inhibitory efficacy was that of strain AS3C1 (83.3%), followed by strains 62 and 86. The broth culture of yeast AS3C1 showed the highest inhibition against isolates 1B3R (1), 1A2R 1.2, 1A3R 1.1, AA and 1A3R (2), with inhibition percentages ranging from 91.5% to 100%. Conversely, the isolates that exhibited the lowest inhibition by the broth culture were 1A1R 2.2 (62.5%) and CB4 (63.2%). For the BC of strain 86, its efficacy was highest against mold CB2 (89.2%), while the least inhibited mold was CB3, with an inhibition percentage of 57.2%. The yeast strain 62 showed an inhibition percentage ranging between 90.4% and 67.5%, with the highest recorded for strain 1A3R (2) and the lowest for CB4. Figure 6, by way of example, shows the inhibitory effect against *A. apis* CB2 by the CP and BC matrices of *M. pulcherrima* 86.

**Figure 7:**
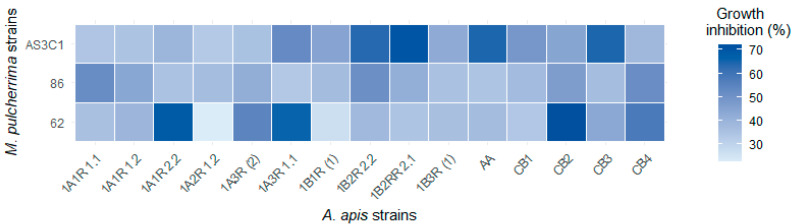
Heatmap showing the percentage inhibition after six days of the fifteen *A. apis* strains caused by the VOCs produced by *M. pulcherrima* AS3C1, 86, and 62 using double-dishes system (DDS).

The results of the inhibitory activity exhibited by the VOCs produced by *M. pulcherrima* are presented graphically in Figure 7 and numerically in Supplementary Table S4. The VOCs with the highest average inhibitory effect were those made by yeast AS3C1 (47.5%), followed by yeasts 86 (44.6%) and 62 (41.1%). Regarding the volatile compounds produced by AS3C1, radial inhibition ranged from 33.2% (1A2R 1.2) to 70.3% (1B2R 2.1). Regarding the VOCs produced by yeast 62, the most inhibited molds were CB2 (72.3%), 1A1R 2.2 (68.6%), and 1A3R 1.1 (65.9%). In contrast, yeast 86 induced inhibitions of 52.2%, 51.8%, and 51.1% against molds 1A1R 1.1, CB4, and 1B2R 2.2, respectively. The findings indicate that the antifungal activity of the VOCs produced by the yeasts is not uniform but varies according to the specific yeast–mold combination.

Despite the extensive literature on the utilization of *Metschnikowia* yeasts as bio-control agents (BCA) against postharvest pathogens, there is no evidence supporting the employment of these yeasts in the management of *A. apis*. The yeasts used in this experiment had been previously characterized for their ability to produce pulcherrimin [84,85]. Pulcherriminic acid is a secondary metabolite naturally produced by several microorganisms, most notably *M. pulcherrima*, which is able to chelate iron ions through a non-enzymatic reaction to form the extracellular red pigment pulcherrimin. The production of this insoluble compound results in the depletion of iron from the environment, making it unavailable for other microorganisms that need it for their growth [67,68,74,106]. The antifungal activity observed in our study can also be attributed to this mechanism [69,107]. In a study by Sipiczki [67], two *M. pulcherrima* isolates were tested for their biocontrol against various fungi, including *Botrytis cinerea*, revealing that strains with lower pulcherrimin production exhibited reduced biocontrol activity. Oro et al. [70] further demonstrated the efficacy of *M. pulcherrima* Disva 267 for the biocontrol of postharvest brown rot of sweet cherries. According to Settier-Ramírez et al. [72], the use of *M. pulcherrima* Y29M has proven effective for post-harvest control of *Penicillium expansum* on apples. Additional antimicrobial mechanisms of *M. pulcherrima* include nutrient competition, biofilm production, and the production of lytic enzymes such as chitinase, like other microorganisms, including lactic acid bacteria [108,109,110,111,112,113]. In a study conducted by Lombardo et al. [73], *M. pulcherrima* MPR3 demonstrated strong biocontrol efficacy against *B. cinerea* and *Erysiphe necator* on table grapes, with antifungal activity attributed to nutrient and space competition, as well as pulcherrimin production.

### 3.4. Identification of Antifungal Volatile Organic Compounds (VOCs)

**Figure 8:**
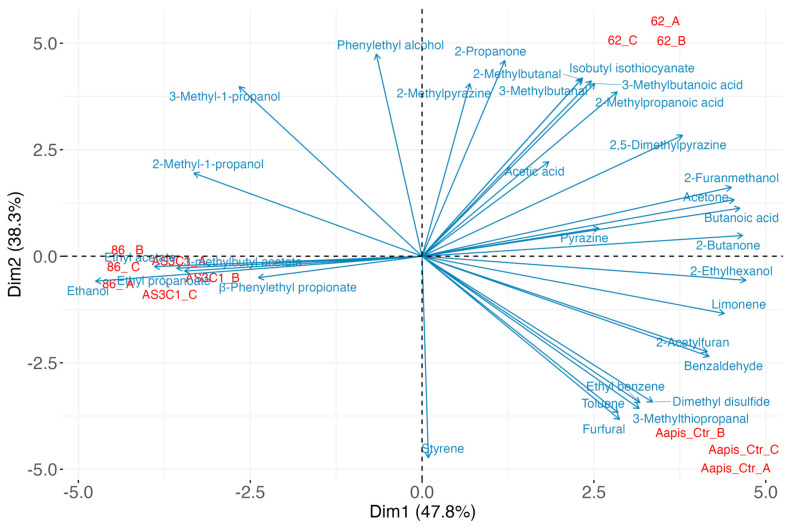
Principal component analysis (PCA) reporting the projection of the samples along the first two principal components (Dim1 and Dim2).

According to several studies, VOCs produced by some microorganisms play a crucial role in inhibiting pathogens, particularly those responsible for post-harvest diseases [109,114]. The variability in the efficacy of different VOCs may be influenced by the specific fungal target. The application of VOCs produced by yeasts, including *M. pulcherrima*, has been extensively studied in the biological control of post-harvest plant pathogenic fungi [115,116,117]. However, there are no reports of specific VOCs, produced by yeasts, for *A. apis* inhibition. The analytical method used made it possible to detect a total of 33 VOCs. Each *M. pulcherrima* strain produced a unique combination of VOCs (Table S5, Supplementary Material). While some compounds were produced by all strains, other compounds were strain specific. The results of PCA (Figure 8) showed that the first two principal components (Dim1 and Dim2) displayed about 86% of the variance.

**Figure 9:**
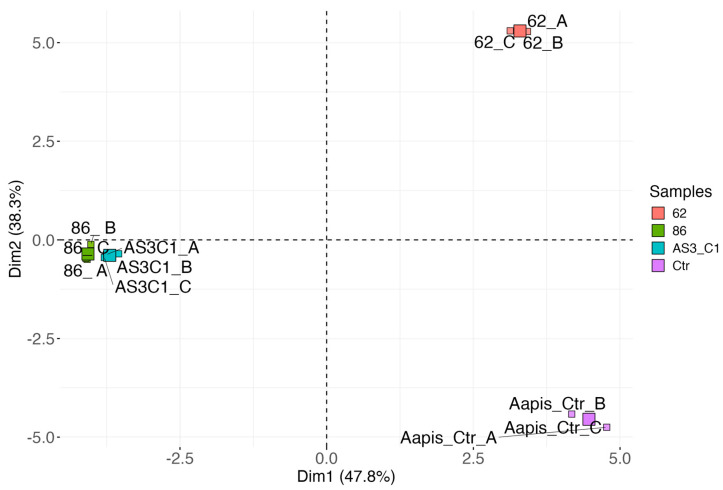
Biplot-PCA reporting the projection of volatile compounds (VOCs) and samples along the first two principal components (Dim1 and Dim2).

Dim1, which accounted for 47.8% of the variance, effectively separated samples 86 and AS3C1 from samples 62 and Aapis_Ctr. Dim2 further distinguished sample 62 from the control (Aapis_Ctr). VOCs mainly responsible for the variation along Dim1 included ethanol, 2-ethylhexanol, 2-butanone, butanoic acid, acetone, 2-furanmethanol, limonene, benzaldehyde, 2-acetylfuran, ethyl acetate, 2,5-dimethylpyrazine, 3-methylbutyl acetate, and ethyl propanoate. Notably, ethanol, ethyl acetate, ethyl propanoate, and 3-methylbutyl acetate were positively associated with Dim1 and with samples 86 and AS3C1 (Figure 9). In contrast, sample 62 was positively correlated with 2-methylbutanal, 3-methylbutanal, isobutyl isothiocyanate, 3-methylbutanoic acid, and 2-methylbutanoic acid, while the control sample (Aapis_Ctr) correlated with 3-methylthiopropanal, dimethyl disulfide, ethyl benzene, toluene, and furfural.

Yeasts exhibit antifungal properties through various mechanisms, including the enhancement of host defenses, competition for nutrients, and the production of antifungal VOCs [118]. Microbial VOCs, which result from various metabolic pathways during the growth of fungi and bacteria, are characterized by low molecular weight (<300 Da) and high vapor pressure (≥0.01 kPa at 20 °C), which is why they evaporate and diffuse easily, making them ideal candidates for agricultural biocontrol applications [71,119,120,121,122]. The capacity of these compounds to exert antimicrobial activity in the absence of direct contact with pathogens is indicative of their potential utility [123]. Referring to this peculiarity, Parafati et al. [124] proposed the use of hydrogel spheres as supports for VOC-producing biocontrol yeasts, opening new avenues for bio-emitters in postharvest packaging.

However, the composition and antifungal properties of volatiles produced by microorganisms are influenced by several environmental and biological factors, including the growth medium, oxygen availability, moisture, temperature, pH, population dynamics, and functional interactions [125]. Among VOCs, esters and alcohols possess antifungal properties and are considered toxic to fungi [116,125]. Antifungal activity of VOCs has been reported to be associated with their functional group [126], and the main mechanism underlying the antifungal effects is the alteration of cell wall and membrane structures, leading to intracellular lysate leakage and oxidative stress induction [118,126,127]. Notably, a mixture of ethanol, 2-methyl-1-propanol, 3-methyl-1-butanol, and 2-phenylethanol altered cell wall structures and resulted in electrolyte leakage in both *B. cinerea* and *A. alternata* [128].

Our findings show that all three *M. pulcherrima* strains produce ethyl acetate, phenylethyl alcohol (2-phenylethanol), 2-methyl-1-propanol, and 3-methyl-1-propanol. Ethyl acetate, synthesized through the reaction of acetic acid with ethanol by yeasts including *M. pulcherrima*, can inhibit the growth of various fruit fungal pathogens and is therefore effective for post-harvest disease management [71,129]. Prior studies have demonstrated that VOCs as alcohols (ethyl alcohol, 3-methyl-1-butanol, and phenylethyl alcohol) and esters (ethyl acetate and isoamyl acetate) produced by *Wickerhamomyces anomalus, M. pulcherrima, Aureobasidium pullulans,* and *Saccharomyces cerevisiae* effectively inhibited postharvest fruit pathogens [123,130,131]. Research by Li et al. [83] highlighted that *M. pulcherrima* T-2 produced several VOCs with antifungal activity against *B. cinerea*, including benzyl alcohol, phenylethyl alcohol, benzaldehyde, 2-ethyl-1-hexanol, acetic acid, octanoic acid, 3-hydroxy-2-butanone, 2,5-dimethylpyrazine, and isoamyl acetate. Among these, 2-phenylethanol exhibited strong antifungal activity, with its mechanism attributed to ROS stress induction and cell membrane alteration [132,133]. Some of the VOCs known to have antifungal activity against plant pathogens include organic acids such as 3-methylbutanoic acid [118], which our tests showed was produced by *M. pulcherrima* 62.

*M. pulcherrima* AS3C1 compared with the other two strains, *M. pulcherrima* 62 and 86, differed in the production of 2-phenethylpropionate. This ester of phenethyl alcohol and propionic acid has shown antifungal activity and has been tested as a pesticide [134,135]. Meanwhile, *M. pulcherrima* 62 differed significantly from other yeasts tested for the production of 3-methylbutanoic acid and isobutyl isothiocyanate among VOCs (Figure 8).

Some of the VOCs are well-documented for their potent antifungal activity against plant pathogens, notably organic acids such as 3-methylbutanoic acid [118]. Additionally, the antifungal efficacy of isothiocyanates against both plant and food-borne pathogens has been extensively reported in the literature [136,137,138,139,140]. Isothiocyanates, derived from glucosinolate hydrolysis in cruciferous plants, are characterized by electrophilic carbon atoms that readily react with cellular thiols, disrupting protein structure and function [141]. These are organic compounds containing the isothiocyanate group RN=C=S. It is thought that this functional group has highly electrophilic carbon atoms, which make it easy to react with thiols in cells, such as cysteine in proteins and low molecular weight thiols (especially glutathione). This change results in the loss of protein structure and function. Isothiocyanate exhibits antifungal activity by targeting cell membrane integrity, cell cycle progression, and oxidative stress [140,142,143]. Isothiocyanates are reported not only to be antifungal but also to affect the growth and survival of some insect larvae [144]. However, several studies have shown that the larvae of some insects have the ability to detoxify isothiocyanates [145] and that no significant effects on honeybee development and mortality were observed in treatment with isothiocyanates against *Nosema ceranae* [146].

Pyrazines, including 2-methylpyrazine, produced by *M. pulcherrima* 86 and 62 (Table S5, Supplementary Materials), are widely studied due to their diverse biological applications. These aromatic hydrocarbons are generally recognized as safe (GRAS) and are commonly found in potatoes, coffee beans, cocoa beans, nuts, beef, and tobacco [147,148]. In addition, pyrazine derivatives continue to attract the attention of several researchers because of their diverse biological applications, such as being used as antifungal agents [149,150].

Finally, the detection of styrene among VOCs in the control sample (Aapis_Ctr) is likely attributable to contamination from plastic materials used during testing, as styrene is commonly emitted from plastics [151,152]. Additionally, certain molds can synthesize styrene from phenylalanine via ammonia-lyase activity followed by cinnamic acid decarboxylation [153,154].

## 4. Conclusions

In recent years, there has been a growing demand for safe and sustainable agricultural products, driving significant interest in biological control agents as viable alternatives to chemical pesticides. Biological control methods, particularly those involving biocontrol agents, offer a promising and environmentally friendly substitute for chemical fungicides, which are increasingly scrutinized due to concerns over food contamination and environmental pollution.

This study represents the first report on the antifungal activity of volatile organic compounds (VOCs) produced by *M. pulcherrima* against *A. apis.* The findings indicate that the antifungal properties of *M. pulcherrima* against *A. apis* may involve multiple mechanisms, including nutrient competition, the secretion of iron-chelating agents (pulcherriminic acid), and the biosynthesis of specific antifungal VOCs.

While this research provides a robust foundation for understanding the yeast’s biocontrol mechanisms, further investigation will be carried out to assess the individual and combined effects of the identified VOCs in controlled experimental settings. Such studies could elucidate potential synergistic interactions that enhance antifungal efficacy against *A. apis*. Furthermore, in the future, it is important to evaluate the effect of *M. pulcherrima* VOCs on the brood of healthy honeybees to demonstrate their safety and identify an application strategy without altering the environmental conditions of the hive and the productivity of honeybee colonies.

Ultimately, this approach may be useful for the development of innovative biocontrol strategies aimed at mitigating chalkbrood disease in honeybee colonies.

## Data Availability

The original contributions presented in this study are included in the article/Supplementary Materials. Further inquiries can be directed to the corresponding authors.

## Supplementary Materials

The following supporting information can be downloaded at https://www.mdpi.com/article/10.3390/jof11050336/s1: Table S1. Fungal isolates list; Table S2. Composition of the culture media used for growth tests of *Ascosphaera apis*; Table S3. BC antifungal activity; Table S4. VOCs antifungal activity; Table S5. VOCs raw data.
