# Supplementary material for: First Report on Antifungal Activity of Metschnikowia pulcherrima Against Ascosphaera apis, the Causative Agent of Chalkbrood Disease in Honeybee (Apis mellifera L.) Colonies
Source: J Fungi (Basel). 2025 Apr 25;11(5):336. doi: 10.3390/jof11050336 (PMC12112871; doi:10.3390/jof11050336)
Supplement: Supplementary file 1 [file jof-11-00336-s001.zip › Table S1_Fungal_isolates_list.pdf]

Journal of Fungi

Table S1. List of the identified *A. apis* strains with corresponding GenBank (NCBI) accession numbers.

Fungal strain ID	Taxonomical identification	Accession number
1A1R 2.2	*Ascosphaera apis*	PV056024
1B3R (1)	*Ascosphaera apis*	PV056025
1A1R 1.1	*Ascosphaera apis*	PV056026
1A3R (2)	*Ascosphaera apis*	PV056027
1B1R (1)	*Ascosphaera apis*	PV056028
1A3R 1.1	*Ascosphaera apis*	PV056029
CB2	*Ascosphaera apis*	PV056030
CB3	*Ascosphaera apis*	PV056031
1A1R 1.2	*Ascosphaera apis*	PV056032
AA	*Ascosphaera apis*	PV056033
CB1	*Ascosphaera apis*	PV056034
1A2R 1.2	*Ascosphaera apis*	PV056035
1B2R 2.2	*Ascosphaera apis*	PV056036
1B2R 2.1	*Ascosphaera apis*	PV056037
CB4	*Ascosphaera apis*	PV056038
