# Supplementary material for: First Report on Antifungal Activity of Metschnikowia pulcherrima Against Ascosphaera apis, the Causative Agent of Chalkbrood Disease in Honeybee (Apis mellifera L.) Colonies
Source: J Fungi (Basel). 2025 Apr 25;11(5):336. doi: 10.3390/jof11050336 (PMC12112871; doi:10.3390/jof11050336)
Supplement: Supplementary file 1 [file jof-11-00336-s001.zip › Table S2. Composition of the culture media used for growth tests of Ascosphaera apis.pdf]

Journal of Fungi

Table S2. Composition of the culture media used for growth tests of *Ascosphaera apis*

Malt Extract Agar (MEA) composition:

	g/L
Malt extract	30.0
Mycological peptone	5.0
Agar	15.0

Potato Dextrose Agar (PDA) composition:

	g/L
Potato infusion	4.0*
Glucose (dextrose)	20.0
Agar	15.0

*infusion from 200 g potatoes

Sabouraud Dextrose Agar (SDA) composition:

	g/L
Mycological peptone	10.0
Glucose (dextrose)	40.0
Agar	15.0

 https://doi.org/10.3390/jof11050336
