# Supplementary material for: First Report on Antifungal Activity of Metschnikowia pulcherrima Against Ascosphaera apis, the Causative Agent of Chalkbrood Disease in Honeybee (Apis mellifera L.) Colonies
Source: J Fungi (Basel). 2025 Apr 25;11(5):336. doi: 10.3390/jof11050336 (PMC12112871; doi:10.3390/jof11050336)
Supplement: Supplementary file 1 [file jof-11-00336-s001.zip › Table S3_BC_antifungal_activity.pdf]

Journal of *Fungi*

MDPI

Table S3. Percentage inhibition of mycelium radial growth (after 6 days on SDA medium) of the *Ascosphaera apis* strains induced by the broth cultures (BC) of *M. pulcherrima* AS3C1, 86 and 62. Values are presented as mean ± SD (n = 3). Different uppercase letters (A-J) within a row and different lowercase letters (a-c) in columns indicate significant differences ($p < 0.05$).

***M. pulcherrima* strains**	*Ascosphaera apis* strains														
	**1B2R 2.1**	**1A3R 1.1**	**1B1R (1)**	**1A2R 1.2**	**AA**	**CB4**	**1A3R (2)**	**1A1R 2.2**	**CB2**	**CB3**	**1B2R 2.2**	**1A1R 1.1**	**CB1**	**1A1R 1.2**	**1B3R (1)**
**AS3C1**	86.3 ± 0.7^{DEa}	93.1 ± 0.8^{BCa}	82.7 ± 0.8^{Fa}	92.3 ± 0.3^{Ca}	95.0 ± 0.6^{Ba}	63.2 ± 0.3^{Hb}	100.0 ± 0.0^{Aa}	62.5 ± 0.9^{Hc}	82.7 ± 0.6^{Fc}	83.0 ± 0.8^{Fa}	72.8 ± 0.8^{Gb}	84.8 ± 0.8^{EFa}	72.5 ± 0.8^{Gc}	87.0 ± 0.7^{Da}	91.5 ± 0.7^{Ca}
**86**	83.9 ± 0.6^{Bb}	84.2 ± 0.8^{Bc}	81.4 ± 0.9^{Cab}	81.7 ± 0.6^{Cc}	74.7 ± 0.8^{Ec}	67.4 ± 0.4^{Ga}	81.7 ± 0.5^{Cc}	76.3 ± 0.6^{DEa}	89.2 ± 0.4^{Aa}	57.2 ± 0.6^{Hc}	75.0 ± 0.5^{DEa}	75.8 ± 0.8^{DEc}	80.9 ± 0.8^{Ca}	71.7 ± 0.6^{Fc}	76.9 ± 0.8^{Dc}
**62**	82.4 ± 0.3^{Dc}	87.1 ± 0.2^{Bb}	80.3 ± 0.5^{Eb}	83.8 ± 0.2^{Cb}	82.4 ± 0.4^{Db}	67.5 ± 0.4^{Ja}	90.4 ± 0.3^{Ab}	70.8 ± 0.2^{Ib}	83.9 ± 0.3^{Cb}	70.1 ± 0.2^{Ib}	72.5 ± 0.3^{Hb}	79.1 ± 0.2^{Fb}	78.3 ± 0.3^{Fb}	77.4 ± 0.4^{Gb}	82.8 ± 0.2^{Db}
