# Supplementary material for: First Report on Antifungal Activity of Metschnikowia pulcherrima Against Ascosphaera apis, the Causative Agent of Chalkbrood Disease in Honeybee (Apis mellifera L.) Colonies
Source: J Fungi (Basel). 2025 Apr 25;11(5):336. doi: 10.3390/jof11050336 (PMC12112871; doi:10.3390/jof11050336)
Supplement: Supplementary file 1 [file jof-11-00336-s001.zip › Table S4_VOCs_antifungal_activity.pdf]

Journal of Fungi

Table S4. Percentage inhibition of radial growth (after 6 days) of the *Ascosphaera apis* strains caused by the VOCs produced by *M. pulcherrima* AS3C1, 86 and 62. The data are presented as mean ± SD (n = 3). Values are presented as mean ± SD (n = 3). Different uppercase letters (A-M) within a row and different lowercase letters (a-c) in columns indicate significant differences ($p < 0.05$).

***M. pulcherrima* strains**	*Ascosphaera apis* strains														
	**1B2R 2.1**	**1A3R 1.1**	**1B1R (1)**	**1A2R 1.2**	**AA**	**CB4**	**1A3R (2)**	**1A1R 2.2**	**CB2**	**CB3**	**1B2R 2.2**	**1A1R 1.1**	**CB1**	**1A1R 1.2**	**1B3R (1)**
**AS3C1**	70.3 ± 0.6^{Aa}	52.8 ± 0.9^{Cb}	45.0 ± 0.4^{Ea}	33.2 ± 0.4^{Gb}	64.7 ± 0.7^{Ba}	38.5 ± 0.3^{Fc}	35.4 ± 0.4^{Gc}	39.4± 0.4^{Fb}	44.7 ± 1.4^{Ec}	64.7 ± 0.6^{Ba}	63.6 ± 0.6^{Ba}	34.5 ± 1.6^{Gb}	48.7 ± 0.6^{Da}	35.0 ± 0.5^{Gc}	42.8 ± 1.9^{Ea}
**86**	34.7 ± 0.7^{JKc}	65.9 ± 0.6^{Ca}	26.7 ± 0.9^{Lc}	22.7 ± 0.3^{Mc}	37.2 ± 0.6^{HIb}	58.4 ± 0.4^{Da}	54.9 ± 0.6^{Ea}	68.6 ± 0.6^{Ba}	72.3 ± 0.2^{Aa}	43.8 ± 0.6^{Fb}	38.1 ± 0.4^{GHc}	36.0 ± 0.5^{IJb}	33.8 ± 0.6^{Kc}	39.2 ± 0.8^{Gb}	36.1 ± 0.5^{IJb}
**62**	41.7 ± 0.8^{Cb}	33.4 ± 0.8^{Fc}	37.1 ± 0.5^{DEb}	36.9 ± 0.7^{DEa}	34.8 ± 0.3^{EFc}	51.8 ± 0.5^{Ab}	41.8 ± 0.5^{Cb}	35.4 ± 0.5^{DEFc}	47.0 ± 1.4^{Bb}	36.9 ± 1.7^{DEc}	51.1 ± 1.1^{Ab}	52.2 ± 1.6^{Aa}	37.4 ± 0.4^{Db}	44.0 ± 1.1^{Ca}	35.6 ± 0.6^{DEFb}
