# Supplementary material for: First Report on Antifungal Activity of Metschnikowia pulcherrima Against Ascosphaera apis, the Causative Agent of Chalkbrood Disease in Honeybee (Apis mellifera L.) Colonies
Source: J Fungi (Basel). 2025 Apr 25;11(5):336. doi: 10.3390/jof11050336 (PMC12112871; doi:10.3390/jof11050336)
Supplement: Supplementary file 1 [file jof-11-00336-s001.zip › Table S5_VOCs_raw_data.pdf]

Journal of Fungi

Table S5. Volatile organic compounds (VOCs) detected by HS-SPME/GC-MS in the double dish sets. Data are mean values of 3 replicates and are expressed as percentages relative to the total peak area of all detected volatile compounds in each sample (RPA %).

Retention time	Compounds	AApis Ctr A	AApis Ctr B	AApis Ctr C	AS3C1_A	AS3C1_B	AS3C1_C	86_ A	86_ B	86_ C	62_A	62_B	62_C
2.247	Acetone	1,91	1,72	1,81	0,10	0,11	0,09	0,44	0,44	0,44	2,29	2,23	2,26
3.289	2-Butanone	1,17	1,12	1,15	0,00	0,00	0,00	0,20	0,26	0,27	1,20	1,20	1,02
4.844	2-Propanone	0,00	0,00	0,00	0,16	0,18	0,15	0,30	0,37	0,29	1,05	1,06	1,08
3.093	Ethyl acetate	0,00	0,00	0,00	8,16	8,83	8,32	27,60	27,44	26,61	1,05	0,96	0,95
4.415	Ethyl propanoate	0,00	0,00	0,00	0,30	0,19	0,21	1,46	1,44	1,45	0,00	0,00	0,00
9.073	3-methylbutyl acetate	0,00	0,00	0,00	0,63	0,59	0,53	2,88	2,89	2,91	0,06	0,05	0,06
32.902	β-Phenylethyl propionate	0,58	0,53	0,51	6,69	6,69	6,81	0,00	0,00	0,00	0,18	0,19	0,18
3.499	2-Methylbutanal	0,00	0,00	0,00	0,00	0,00	0,00	0,00	0,00	0,00	1,45	1,93	1,69
3.572	3-Methylbutanal	0,24	0,15	0,20	0,00	0,00	0,00	0,06	0,08	0,04	5,26	5,20	5,24
20.493	3-Methylthiopropanal	0,28	0,21	0,25	0,00	0,00	0,00	0,00	0,00	0,00	0,00	0,00	0,00
22.603	Benzaldehyde	0,88	0,81	0,84	0,06	0,09	0,06	0,08	0,08	0,08	0,32	0,37	0,30
3.963	Ethanol	0,46	0,45	0,45	19,02	18,96	18,32	21,68	21,21	20,98	0,00	0,00	0,00
6.496	Toluene	1,53	1,51	1,50	0,26	0,27	0,23	0,58	0,56	0,57	0,33	0,39	0,31
8.152	2-Methyl-1-propanol	0,17	0,20	0,18	1,36	1,26	1,24	3,16	3,13	3,14	1,69	1,51	1,61
12.093	3-Methyl-1-propanol	11,69	11,23	11,96	36,45	36,55	35,45	36,41	38,92	37,70	40,83	40,22	40,99
21.437	2-Ethylhexanol	0,34	0,33	0,34	0,03	0,06	0,04	0,09	0,06	0,04	0,28	0,23	0,26
33.6	Phenylethyl alcohol	1,78	1,68	1,67	14,88	15,26	14,99	16,00	16,49	16,28	28,23	28,25	28,72
12.152	Pyrazine	1,04	1,31	1,18	0,00	0,00	0,00	0,98	1,30	1,35	1,20	1,33	1,32
14.064	2-Methylpyrazine	0,00	0,00	0,00	0,00	0,00	0,00	0,33	0,44	0,22	0,49	0,66	0,58
15.854	2,5-Dimethylpyrazine	0,49	0,57	0,58	0,33	0,32	0,28	0,22	0,34	0,33	0,93	0,85	0,89

Table S5. Continued

21.069	Acetic acid	0,40	0,37	0,39	0,53	0,53	0,45	0,25	0,21	0,18	0,69	0,43	0,56
24.467	2-Methylpropanoic acid	0,14	0,11	0,13	0,00	0,00	0,00	0,00	0,00	0,00	0,99	1,05	0,92
26.251	Butanoic acid	0,53	0,50	0,52	0,00	0,00	0,00	0,00	0,00	0,00	0,53	0,71	0,62
27.417	3-Methylbutanoic acid	0,15	0,10	0,10	0,02	0,04	0,02	0,04	0,04	0,04	1,96	1,66	1,63
20.834	Furfural	0,47	0,44	0,41	0,03	0,04	0,03	0,04	0,04	0,04	0,00	0,00	0,00
22.047	2-Acetylfuran	0,84	0,86	0,85	0,00	0,00	0,00	0,12	0,16	0,12	0,36	0,35	0,30
26.810	2-Furanmethanol	0,61	0,50	0,55	0,11	0,18	0,13	0,12	0,12	0,12	0,74	0,72	0,73
7.511	Dimethyl disulfide	7,28	7,20	7,26	0,00	0,00	0,00	0,07	0,09	0,05	0,53	0,33	0,43
9.162	Ethyl benzene	1,65	1,43	1,54	0,41	0,41	0,36	0,69	0,56	0,63	0,58	0,53	0,51
11.467	Limonene	0,76	0,60	0,68	0,19	0,19	0,17	0,33	0,27	0,26	0,42	0,53	0,48
13.767	Styrene	48,56	48,21	48,37	26,05	25,28	25,12	32,69	32,15	33,15	4,58	4,73	4,79
15.848	Isobutyl isothiocyanate	0,00	0,00	0,00	0,00	0,00	0,00	0,00	0,00	0,00	3,22	3,49	3,35
37.8	γ-Decanolactone	1,10	1,08	1,09	0,13	0,13	0,11	0,25	0,25	0,25	0,99	0,94	0,96
